# The Role of Online Peer Support Groups in the Recovery of Romance Fraud Victims

**DOI:** 10.1093/geroni/igaf122.658

**Published:** 2025-12-31

**Authors:** Jennifer Lawrence

**Affiliations:** FightCybercrime, Boston, Massachusetts, United States

## Abstract

Romance frauds lead to emotional, psychological, and financial distress that last for years after the scam concludes. This presentation examines the role of online peer support groups in helping victims recover after a scam. Peer support programs that address a victim’s loneliness and shame, while also educating participants in scammer tactics and cyber security best practices can help victims rebuild their lives across multiple aspects. This holistic approach helps victims through recovery and equips them with the tools needed to respond to future scams. In 2024, a FightCybercrime post-peer support group survey of 67 participants examined the factors influencing victims’ initial engagement with scammers. Loneliness emerged as the most significant factor, followed by a lack of awareness of scam sophistication. In the survey, participants noted that the peer support group helped them recognize how scammers take advantage of these vulnerabilities. Also, 87% of those surveyed stated that the peer support group provided valuable resources to address these challenges. Finally, qualitative responses described the benefits of shared experiences, emotional validation, and information on cyber security best practices to help avoid future scams. These findings emphasize the role of peer support in mitigating the impact of romance scams and preventing revictimization.

